# Economic crisis and smoking in health professionals in Greece

**DOI:** 10.1186/1617-9625-12-S1-A23

**Published:** 2014-06-06

**Authors:** Eleni Litsiou, Aikaterini Tsoutsa, Vasiliki Saltagianni, Dimos Fotopoulos, Stavroula Kolokytha, Spyridon Zakynthinos, Paraskevi Katsaounou

**Affiliations:** 1 Pulmonary department-ICU, Εvaggelismos Hospital, Athens, 10676, Greece; 2 OKANA, Addiction Unit, Rethymnon, 74100, Greece

## Background

Despite the reduction of consumer disposable income due to the economic crisis which negatively affected tobacco (Euromonitor, 2012), the proportion of smokers in Greece is still the highest among all European Countries (42.0%). This percentage is even high in medical students (35.0%) and professionals [1,2].

## Materials and methods

In our study, we investigated how occupational factors and economic crisis affect smoking in health professionals in Evaggelismos Hospital (the largest in Greece). Our questionnaires included smoking history, demographic factors, working data, intention to quit and 4DSQ (measuring depression anxiety and related psychosomatic symptoms). In our sample of 500 participants (men/women=1:4, 37.0% medical doctors, 58.0% nurses), 49.0% were smokers. Initially, we explored work factors affected by crisis. The amount of work has increased for 45% of workers with a parallel decrease of 10.0-20.0% in the income of 90.0%. After collecting the questionnaires we distributed informative leaflets about our Smoking Cessation Outpatient Clinic (SCC).

## Results

Taking into account that a smoker (1 pack/d) spends on average 10.0-20.0% of his salary for smoking, it is a paradox that only 25.0% of them report an intention to quit. This could be explained because smokers addicted to nicotine use smoking for handling stress and depression. Consequently, our SCC focused in informing the staff that nicotine is a stimulant agent and thus is wrongly interpreted as relaxant. We initiated a cognitive intervention-motivational coaching program in order to stop this vicious circle. Namely to dissociate smoking used for craving and alleviation of withdrawal symptoms from real-life stress. After being informed, 30.0% of smokers intend attempting smoking cessation in our clinic.

## Conclusions

Further information and intervention programs are necessary so that smokers are convinced that nicotine is a stimulant agent and thus is wrongly interpreted as relaxant.

## References

[B1] Caleyachetty A Lewis S McNeill A Leonardi-Bee J Struggling to make ends meet: exploring pathways to understand why smokers in financial difficulties are less likely to quit successfully Eur J Public Health 2012 22 1 41 8 22294784 10.1093/eurpub/ckr199 PMC3269295

[B2] Vardavas CI Bouloukaki I Linardakis MK Tzilepi P Tzanakis N Kafatos AG Smoke-free hospitals in Greece: Personnel perceptions, compliance and smoking habit TobInduc Dis 2009 31 5 1 8 10.1186/1617-9625-5-8 PMC2669059 19335886

